# Neural network models for DMT-induced visual hallucinations

**DOI:** 10.1093/nc/niaa024

**Published:** 2020-12-12

**Authors:** Michael M Schartner, Christopher Timmermann

**Affiliations:** n1 Département des Neurosciences Fondamentales, Université de Genève Rue Michel Servet 1 CH-1211 Geneva Switzerland; n2 Department of Medicine, Centre for Psychedelic Research, Division of Brain Sciences, Imperial College London, UK The Commonwealth Building, The Hammersmith Hospital, Du Cane Road, London W12 0NN; n3 Computational, Cognitive and Clinical Neuroscience Laboratory (C3NL), Department of Medicine, Imperial College London, UK Burlington Danes Building Hammersmith Hospital Du Cane Road London W12 0NN

**Keywords:** computational modelling, imagery, perception, pharmacology

## Abstract

The regulatory role of the serotonergic system on conscious perception can be investigated perturbatorily with psychedelic drugs such as N,N-Dimethyltryptamine. There is increasing evidence that the serotonergic system gates prior (endogenous) and sensory (exogenous) information in the construction of a conscious experience. Using two generative deep neural networks as examples, we discuss how such models have the potential to be, firstly, an important medium to illustrate phenomenological visual effects of psychedelics—besides paintings, verbal reports and psychometric testing—and, secondly, their utility to conceptualize biological mechanisms of gating the influence of exogenous and endogenous information on visual perception.

## Introduction

The framework of predictive coding states that the human brain generates a model of the world by constantly combining prior beliefs with sensory information (Friston 2018). The resulting model is partially consciously perceived and subject to report. Each experience depends on a balanced weighting of prior and sensory information, a balance that can be disturbed by classical psychedelics which act primarily via the serotonergic system (Jacobs and Trulson 1979). Inspired by the usage of deep convolutional neural networks to model psychedelic hallucinations (Mordvintsev *et al.* 2015; Suzuki et al. 2017) and increasing evidence on the role of the serotonergic system in gating sensory information (Azimi *et al.* 2020), we suggest two recent generative deep convolutional neural network architectures to illustrate the perturbation of the balanced integration of sensory and prior information associated with visual perception.

We exemplify a psychedelic perturbation via N,N-Dimethyltryptamine (DMT), a hallucinogen known for inducing some of the most vivid and unique forms of visual imagery (i.e. hallucinations) known to science, which can be reliably elicited during eyes-closed conditions (Szara 1956; Strassman *et al.* 1994). Descriptions of DMT-induced changes in conscious perception can be found in answers to systematic questionnaires (Timmermann *et al*. 2018a), phenomenological research (Shanon 2002a, b), anecdotal evidence (Erowid Center erowid’s Experience Vaults) and paintings featured in ‘visionary art’ (Grey 1990; Luna and Amaringo 1999). The striking changes in visual perception caused by DMT (commonly described as ‘immersive’ forms of visual imagery), the short duration of effects (5–20 min) and the low health risk qualify this substance as a well-controlled perturbational tool for the study of conscious perception (Timmermann et al. 2019). Although it is known that DMT’s psychedelic effects result from the molecule binding to various serotonin receptor types, as confirmed behaviourally in both humans (Valle et al. 2016) and mice (Keiser *et al.* 2009), it remains an open question how the molecule perturbs the balanced integration of sensory and prior information in conscious perception.

Deep convolutional neural network architectures are being used as generative models to produce increasingly realistic images, showing e.g. faces, bedrooms or cars that do not actually exist but look deceivingly real (Karras *et al.* 2019). The underlying convolutional network architecture can be seen as a detailed model of the visual processing system in the mammalian brain, as has been shown by matching specific network layers with brain regions in monkeys (Rajalingham *et al.* 2018) and humans (Grossman *et al.* 2019). We present the output of two deep convolutional neural network architectures resulting in visual features reminiscent of descriptions of psychedelic-induced visual imagery. Firstly, using a generative model designed to produce realistic images of human faces (Karras *et al.* 2019), we show the impact of perturbing the noise input of the model and discuss a potential biological interpretation for the omission of noise. Noise is usually added to all levels of the model to produce most realistic output and its omission results in a painterly, smooth version of the generated images, in line with descriptions of imagery reported by people under the influence of DMT. Importantly, this generative model illustrates further how sparse latent information about visual scenes may result in rich conscious experiences, using the ventral visual system in the mammalian brain as a ‘canvas’ for perception or imagination (Pearson 2019). Secondly, we discuss a convolutional network for style transfer – i.e. changing a given ‘content’ image to resemble, in style only, a ‘style’ image (Gatys *et al.* 2016) – which results in images consistent with visual depictions of beings (or ‘entities’) featured in DMT-induced forms of ‘visionary art’ (Grey 1990; Luna and Amaringo 1999). The influence of style onto a given content image can be regulated with a parameter when training the model, which one might explore as a DMT-dose-dependent visual distortion.

The proposed model interpretations are speculative examples intended to illustrate the potential of generative deep neural networks to create visual output in line with psychedelic phenomenology. Such models not only have the potential utility of being a most accurate medium to illustrate visual effects of psychedelics but also to conceptualize potential biological mechanisms of the balanced integration of exogenous and endogenous information into conscious experience.

## NVIDIA’s Face Generator with Noise Perturbations as a Model for DMT’s Effect

NVIDIA’s generative model (Karras *et al.* 2019) consists of an 18-layer feed-forward convolutional neural network, fine-tuned with adversarial network techniques to generate highly realistic images of human faces. Adversarial training of a generative model consists of two networks, a generator and a ‘critic’. The generator network synthesizes an image from latent activity and the ‘critic’ evaluates the distance of this image from the distribution of the training set. Both networks can be trained via back-propagation to improve their performance, assessed via metrics such as the Frechet Inception Distance, measuring the distance between feature vectors (i.e. activity in late but not final network layers) of real and generated images (Karras *et al.* 2017).

**Figure 1.**
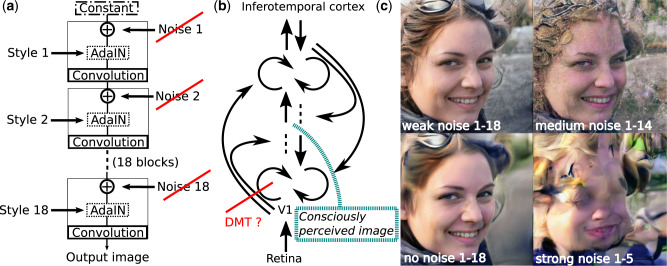
NVIDIA’s generative model with noise perturbation and analogous hypothesized brain mechanism. (**a**) NVIDIA’s styleGAN architecture (Karras *et al.* 2019) consists of 18 blocks, each processing information by adding style input – 512 numbers which were transformed via a trained extra network (not shown), a learned affine transformation and then influence the information stream via adaptive instance normalisation (AdaIN) at each block. Furthermore, scaled noise is independently added before each convolution. (**b**) Sketch of the recurrent information flow, from the retina to a consciously perceived image in the human brain with some of the information flow being blocked by DMT, hypothetically corresponding to the omission of noise in NVIDIA’s generative model. (**c**) The top left face was generated using NVIDIA’s StyleGAN (Karras *et al.* 2019) with default parameters, including weak noise being added to all layers, resulting in realistic output. When removing the noise input completely, the face appears smoother, in line with frequent reports of DMT experiences about ‘cleaned-up’ scenes, shown in the bottom left image. Two additional images illustrate other noise perturbations of the model. The upper-right one with noise input increased by a factor of 10 in amplitude and applied to all but the last four layers. The lower right one shows severe image distortions as a result of strong noise (factor 40) applied to the first five layers.

The output image of the trained generating network is determined by a ‘content’ vector of 512 numbers that influence the input to each convolution, via an extra non-linear multi-layer network and subsequent affine transformations. Different parts of the content vector determine different classes of features of the portrait image, from coarse ones such as face proportions and expression to fine ones such as skin and hair colour. Figure 1a shows a schematic of the architecture developed by Karras *et al.* (2019).

Besides the content vector, the generated output image is further influenced by noise that is added directly to each pixel before each convolution. The omission of this noise results in fewer fine-details in the generated image, such as freckles or individual hair, and an overall smoother look, as shown in Fig. 1c, when comparing the top left image (weak noise added to all layers, 1–18, designed to create the most realistic images) with the bottom left image (complete omission of noise). The resulting absence of image details, being a ‘cleaned-up’ version of the scene, can be widely found in verbal and artistic depictions of the visual effects induced by DMT. Contents of DMT experiences often either consist of low-level features (e.g. geometrical patterns) alone, or complex scenes composed of simple visual motifs (Grey 1990; Strassman 1996; Shanon 2002b). Noticeably, DMT is known for inducing visual imagery, which is able to compete with (and at high doses completely ‘overlay’ over) visual imagery triggered by the external environment, often resulting in confusing experiences, in particular in eyes-open conditions (Strassman 2001).

Unlike in the decoding step of an autoencoder where latent activity in one layer entirely determines the output, the starting activity of NVIDIA’s generative network is constant and the output image is influenced by style and noise input at each layer. The activity in early layers might be interpreted biologically as activity in the inferotemporal cortex (IT) [a compact representation of a visual scene (Kornblith and Tsao 2017; Grossman *et al.* 2019) at the final stage of the ventral visual stream], while the output layer may correspond to more primary visual areas early in the ventral stream (Fig. 1b). That is, the ventral visual system is used top-down for the generation of a consciously perceived image, in line with literature on mental imagery, stating that the visual system is a ‘canvas’ that can either be used by perception or imagination (Pearson 2019). The details of the image depend on the network weights (which we can interpret as endogenous prior information), the input of style in each layer (which can be interpreted either as endogenous, for imagination, or exogenous for perception) and, to a lesser extent, to noise input. That noise input may be interpreted as spontaneous activity in visual areas such as V1, as it was shown that in mice 5-HT2a receptors are involved in ‘powerful scaling of ongoing and evoked components of population activity in V1’ (Azimi *et al.* 2020), which may thus regulate indirectly the exogenous influence on perception.

This is, however, only one interpretation to illustrate the potential of this generative model in producing visual effects reminiscent of those brought about by DMT by manipulating the noise input, while, at the same time, conceptualizing biological mechanisms. In order to illustrate the range of possible noise manipulations of the model, we further show in Fig. 1c, the visual effects of additional noise perturbations, which seem, however, less suitable to model visual effects of DMT. When increasing the noise input amplitude by a factor of 10 (called medium noise in Fig. 1c) and adding this noise to all but the last four layers, the output image becomes less realistic and markedly changed in style. Increasing the noise input amplitude by a factor of 40 and adding it only to the first five layers distorts the image dramatically, with the network hardly being able to stabilize on a face at all (strong noise in Fig. 1c). There are further weak noise manipulations of this network reported in Karras *et al.* (2019) with comparably subtle effects on style.

## Style-Transfer Network with Style Image as Prior

Creating visual effects that resemble those reported by people under the influence of DMT can further be achieved using style-transfer networks. These architectures take a style image and a content image as input and create a stylized version of the content image. This was originally achieved by *Gatys et al.* (2016) using a deep convolutional neural network—trained for image classification on a large training set of natural images—and identifying the activation in deeper layers as encoding content while it was found that the inner product of feature maps combined across several intermediate layers (a Gram matrix) produces a scale-invariant representation of the style of an image. Given two images, a content-loss function can be defined for a given layer as the root-mean-square difference in the activation in that layer caused by the two different images, and a style-loss function as a normalized root-mean-square difference in their style-representing Gram matrices. Using back-propagation, this weighted sum of the content-loss function and the style-loss function allows to change pixels in a random image such that it reflects the style of one image and the content of the other.

**Figure 2.**
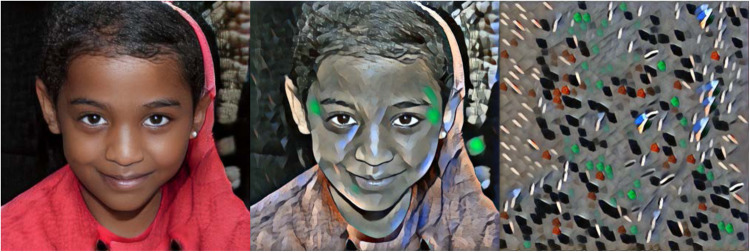
Example output of a style-transfer network. The portrait image of a non-existing child [generated using NVIDIA’s face generator (Karras *et al.* 2019)] was stylized via a style-transfer deep neural network (Fast-Neural-Style Pytorch Implementation for Artistic Style Transfer), shown in the middle. The used style was an image of an abstract painting by Udnie. The stylized image may resemble a stylized world view that people report under the influence of DMT in eyes-open conditions at moderate doses. The right-most image shows the output of the style-transfer network when training the weights with a large bias on style, resulting in a near-complete overwriting of the content image and possibly modelling DMT-effects at high doses. These two examples illustrate a modelling of dose-dependent visual effects via changes in style.

We used a fast approximation (Johnson *et al.* 2016; Fast-Neural-Style Pytorch Implementation for Artistic Style Transfer) of the method by Gatys *et al.* with visually similar results. Figure 2 shows a portrait image (generated with NVIDIA’s face generator) as a content image on the left, stylized using a painting by the artist Udnie, in the middle. The stylized image displays the potential to resemble certain DMT-induced hallucinations, as described in Grey (1990) and Luna and Amaringo (1999).

The general anatomical interpretation of this style-transfer architecture is also the ventral visual stream, Fig. 1b, with the higher layers corresponding to the IT, proposing that the feedback process is part of creating the perceived image in the brain, in line with the notion that vision is an active process, where both, top-down and bottom-up signals are integrated into a consciously perceived image (Gilbert and Li 2013). The activation in later network layers that encode content may be seen as activity in IT, compactly encoding the content of a scene, while activity in earlier layers encodes details (the style) of the image. The weights of the model are fixed by training on a large set of natural images and the influence of the style has to be set by one scalar before training. That is, a content image is changed according to the style, whose intensity can be set before training. A possible biological interpretation is to see the style as endogenous prior information – encoded in the weights of the network – while the external sensory information – the input image – is kept constant.

Interpreting the content to reflect veridical, exogenous information while the style is endogenous, can be motivated by subjective accounts of DMT experiences. At small or medium doses, reports of DMT-induced perceptual changes with eyes open are often reported as being simple geometric approximations of veridical scenes, more reflecting changes in style rather than content (the actual scene). That is, the ‘broad strokes’ of the perceived scene remain unchanged while the style (amount of detail) is changed. However, phenomenological reports about high doses of eyes-open DMT experiences contain descriptions of perceiving detailed scenes, overlaying on top of the external veridical scene (often called ‘breakthrough’ experience). This would speak in favour of shifts in both endogenous content and endogenous style, similar to dream imagery and eyes-closed imagery associated with DMT administration.

To further illustrate the potential of mimicking DMT-dose-dependent visual distortions, the right-most image in Fig. 2 shows the results of a network trained with a very high weighting for style [content-weight set to 1e5 and style-weight to 1e12 using (Fast-Neural-Style Pytorch Implementation for Artistic Style Transfer), resulting in nearly completely overwriting the content of the input image.

## Discussion

Using two deep convolutional network architectures, we pointed out the potential to generate changes in natural images that are in line with subjective reports of DMT-induced hallucinations. Unlike human paintings of psychedelic hallucinations—the traditional way to illustrate psychedelic imagery—using well-defined deep network architectures allows to draw parallels to brain mechanisms, in particular with respect to a perturbed balance between sensory information and prior information, mediated by the serotonergic system.

In our first model, NVIDIA’s generative model StyleGAN (Karras *et al.* 2019), we show how perturbation of the noise input can lead to image distortions reminiscent of verbal reports from controlled experiments in which DMT has been administered (Timmermann *et al.* 2019). In particular, the omission of noise leads to a smoother, painterly look of the images, illustrating a potential hypothesis that can be conceptualized with such models: as a 5-HT2A receptor agonist, DMT induces a state in which environmental (i.e. exogenous) sensory information is partially blocked—gated by the inserted noise—and system-internal (endogenous) signals are influencing conscious imagery more strongly. Contents of immersive imagery experienced in eyes-closed conditions during DMT administration would thereby correspond to the system’s prior information for the construction of a consciously perceived scene.

Our second model, the style-transfer network architecture, allows us to depict nearly any report of a visual hallucination – assuming one can find a matching content and style image. A possible neural interpretation here is a re-weighing of exogenous and endogenous information caused by DMT, e.g. weakening the sensory information (content image) while increasing the style prior, leading to a simplified depiction of a scene. We hereby add to Google’s ‘deepdream’ results, where a network trained to classify images can be used to backpropagate activation in a certain layer to pixels of a content image (Mordvintsev *et al.* 2015; Suzuki *et al.* 2017), showing patterns resembling those drawn by people that experienced psychedelic states, in particular when recurrently back-propagating pixel changes in accordance with a certain activation pattern in deeper layers.

The amount of possible visual scenes that can be generated with this deep style-transfer network is only bound by the number of style and content images one can find. This generality may be seen as an advantage when conceiving deep neural network models as an important medium to illustrate phenomenological effects of psychedelics, besides illustrations and subjective reports. However, more refined psychometric questionnaires and phenomenological methods (Petitmengin 2006) would be needed to tweak model outputs towards most accurate depictions of common visual effects of DMT. This might be initially achieved by asking participants of DMT experiments to select the most appropriate style-transfer output images from galleries such as deepart (Leon Gatys and Bethge). The model’s merit in terms of biological plausibility lies in the adjustable influence of the two components—style and content—that may be mapped to endogenous and exogenous information, respectively, with the adjustment of the perturbation to reflect the DMT-dose.

With both models, we suggest in particular the suppression of exogenous sensory signals following DMT administration, in line with a study showing that activity very early in the visual stream, in retinal neurons, is suppressed by DMT (Heiss *et al.* 1973). This would appear inconsistent with evidence indicating that psychedelic effects are associated with reductions of top-down and increases in bottom-up signals (Alonso *et al.* 2015; Timmermann *et al*. 2018b; Carhart-Harris and Friston 2019; Alamia *et al.* 2020). This contradiction can be resolved when endogenous activity (i.e. not related to external input) stemming from visual areas is conceptualized as endogenously originating bottom-up activity, as opposed to that of endogenous top-down priors. The importance of primary visual areas for mental imagery without exogenous information has been shown in studies using non-pharmacologically enhanced imagination only (Pearson 2019). This conceptualization is consistent with pervasive reports of perceiving geometrical patterns during psychedelic experiences, which may reflect the anatomical structure of the visual cortex as mathematical modelling suggests [see early phenomenological work on geometrical ‘form constants’ encountered in psychedelic experiences (Klüver 1942) and mathematical modelling of these form constants (Bressloff *et al.* 2001)]. The reported appreciation of novelty regarding these experiences and apparent absence of connection with participants’ previous forms of semantic knowledge or biographical memories [especially prevalent during DMT experiences (Strassman 2001)] is consistent with the idea that these forms of visual experiences represent an increased influence of endogenous prediction errors (usually considered as bottom-up input), incoming from within the visual system, resulting in the updating of top-down priors. These updated priors possibly account for some of the long-term effects associated with psychedelic experiences on personality, brain function and brain anatomy (MacLean *et al.* 2011; Bouso *et al.* 2015; Erritzoe *et al.* 2018; Barrett *et al.* 2020).

Research on image encoding in IT suggests that ‘the computational mission of IT face patches is to generate a robust, efficient, and invariant code for faces, which can then be read-out for any behavioural/cognitive purpose downstream’ (Kornblith and Tsao 2017). The latent information entering the NVIDIA generative model may thus be interpreted as activity in IT and the output image as the consciously perceived scene, constructed during the read-out by other cortical areas. How this read-out creates an experience is at the heart of the mind-body problem and we suggest that modelling the effects of DMT on the balance between exogenous and endogenous information may provide experimentally testable hypotheses about this central question of consciousness science. For the example of compact representations of visual scenes in IT, this is especially relevant, since in this brain region the density of 5-HT2A receptors [primarily associated with psychedelic effects, however, not solely (Barker 2018)] is particularly high (Beliveau *et al.* 2017).

The serotonergic system plays a key role in the balancing between endogenous and exogenous information involved in the construction of conscious experience, as the perturbation by psychedelic molecules such as DMT shows. This balancing is heavily mediated by the interplay between 5-HT1A and 5-HT2A receptors (Nichols 2016; Azimi *et al.* 2020). Although serotonin receptors can be found across the cortex (Beliveau *et al.* 2017), specific mechanisms (at different spatial scales) underlie the effects of psychedelics in perception (Nichols 2004). Here we added a cognitive perspective on these systems neuroscience approaches to identify mechanisms of combining endogenous and exogenous information into conscious experience.

*Conflict of interest statement*. None declared.
